# Human vs. Machine Learning Based Detection of Facial Weakness Using Video Analysis

**DOI:** 10.3389/fneur.2022.878282

**Published:** 2022-07-01

**Authors:** Chad M. Aldridge, Mark M. McDonald, Mattia Wruble, Yan Zhuang, Omar Uribe, Timothy L. McMurry, Iris Lin, Haydon Pitchford, Brett J. Schneider, William A. Dalrymple, Joseph F. Carrera, Sherita Chapman, Bradford B. Worrall, Gustavo K. Rohde, Andrew M. Southerland

**Affiliations:** ^1^Department of Neurology, University of Virginia, Charlottesville, VA, United States; ^2^Department of Electrical and Computer Engineering, University of Virginia, Charlottesville, VA, United States; ^3^Department of Public Health Sciences, University of Virginia, Charlottesville, VA, United States; ^4^Department of Neurology, University of Pittsburgh, Pittsburgh, PA, United States; ^5^Department of Neurology, University of Michigan, Ann Arbor, MI, United States; ^6^Department of Biomedical Engineering, University of Virginia, Charlottesville, VA, United States

**Keywords:** cerebrovascular disease, stroke, infarction, access to care, diagnostic test, computer vision, machine learning

## Abstract

**Background:**

Current EMS stroke screening tools facilitate early detection and triage, but the tools' accuracy and reliability are limited and highly variable. An automated stroke screening tool could improve stroke outcomes by facilitating more accurate prehospital diagnosis and delivery. We hypothesize that a machine learning algorithm using video analysis can detect common signs of stroke. As a proof-of-concept study, we trained a computer algorithm to detect presence and laterality of facial weakness in publically available videos with comparable accuracy, sensitivity, and specificity to paramedics.

**Methods and Results:**

We curated videos of people with unilateral facial weakness (*n* = 93) and with a normal smile (*n* = 96) from publicly available web-based sources. Three board certified vascular neurologists categorized the videos according to the presence or absence of weakness and laterality. Three paramedics independently analyzed each video with a mean accuracy, sensitivity and specificity of 92.6% [95% CI 90.1–94.7%], 87.8% [95% CI 83.9–91.7%] and 99.3% [95% CI 98.2–100%]. Using a 5-fold cross validation scheme, we trained a computer vision algorithm to analyze the same videos producing an accuracy, sensitivity and specificity of 88.9% [95% CI 83.5–93%], 90.3% [95% CI 82.4–95.5%] and 87.5 [95% CI 79.2–93.4%].

**Conclusions:**

These preliminary results suggest that a machine learning algorithm using computer vision analysis can detect unilateral facial weakness in pre-recorded videos with an accuracy and sensitivity comparable to trained paramedics. Further research is warranted to pursue the concept of augmented facial weakness detection and external validation of this algorithm in independent data sets and prospective patient encounters.

## Introduction

Inaccurate detection of common neurologic signs, such as facial weakness, can lead to delays in diagnosis and treatment for a variety of neurological diseases, particularly time-sensitive conditions such as stroke. For instance, emergency medical service (EMS) providers may fail to detect stroke in over half of cases, even when using standard prehospital stroke detection screening tools such as the Cincinnati Prehospital Stroke Scale (CPSS) (1, 2). The CPSS and other screening tools rely heavily on provider experience and training to accurately hone the identification of neurologic deficits, which is most challenging for non-neurologist providers (3). Additionally, neurologic deficits are not easily quantifiable and therefore their interpretation is highly subjective. In one study, paramedics failed to identify facial weakness in 17% of stroke patients and incorrectly interpreted facial weakness as present when it was absent in an additional 33% of cases (4). In a study of over 8,000 raters of the NIH Stroke Scale (NIHSS), including neurologists, nurses, and emergency providers, facial weakness had the second poorest agreement (0.25) of all the scale items (5). Weak inter-operator variability contributes to the wide range of CPSS stroke scale sensitivity and specificity observed in the prehospital setting (1, 6, 7). As a result, many stroke patients go unrecognized or are inappropriately triaged delaying or missing the opportunity for timely acute stroke treatment with thrombolysis. Concomitantly, common stroke mimics are often over triaged unnecessarily expending emergency resources.

Most current, the challenge of stroke screening in the field has been accentuated in the endovascular era, in which the accurate detection of large vessel occlusion (LVO) stroke could inform triage to a thrombectomy capable center (8, 9). Thus, numerous second generation LVO stroke scales have been derived and internally validated but lack consistent performance and generalizability across multiple regions. Additionally, no single scale has demonstrated proven superiority and further external validation and accuracy in real world practice is needed (10).

Computer vision analysis through machine learning has the potential to enhance clinical diagnosis of visually observable diseases, such as diabetic retinopathy and skin cancer (11). Since stroke is a clinical diagnosis reliant on visually observable neurologic signs, we believe that AI can be developed to augment the detection of stroke through recognition and differentiation of focal deficits. Specifically in regards to facial analysis, machine learning algorithms can differentiate between deliberate and spontaneous smiles by analyzing distinct patterns of facial muscle activation (12). Similarly, machine learning can identify subtle facial asymmetry in expressions suggestive of negative emotional valence (13).

Given that gross facial asymmetry is the hallmark of localized facial weakness with impaired muscle contraction. In the current study, we aimed to develop a machine learning algorithm to identify pathological facial weakness using computer based video analysis. Specifically, we hypothesized that a machine learning algorithm can detect asymmetric facial weakness with a similar or better accuracy than trained paramedics.

## Methods

### Standard Protocol Approvals, Registrations, and Patient Consents

All study procedures were in alignment with the Declaration of Helsinki and approved by the institutional review board of the University of Virginia (#20021), which waived the need for informed consent as retrieved videos came from open access (public) repositories.

### Convenient Population Sample

Videos voluntarily submitted by people demonstrating unilateral facial weakness were conveniently collected from open access repositories such as YouTube and Google (14). Videos that contained only one individual with the same individual smiling normally with or without unilateral facial weakness were eligible for inclusion in this study. Videos of people smiling were collected to assure the possibility for an assessment of the presence of pathological asymmetry. There were no additional exclusion criteria in order to achieve a wide range of unilateral facial weakness presentations mirroring real life encounters. As an exploratory proof of concept study, a formal sample size was not estimated prior to sampling videos to be rated.

### Reference Standard

There is no higher gold standard for facial weakness detection other than by clinical assessment. Since vascular neurologists receive specialized training in the accurate and rapid diagnosis of stroke of which facial weakness is a common sign, we assumed that vascular neurologists are the best candidate to be the “ground truth” for detecting the presence or absence of pathological unilateral facial weakness for the purposes of this study. Thus three board-certified vascular neurologists blinded to the type of video independently rated each one denoting the presence or absence of facial weakness.

The study employed a rating scale similar to a Likert scale in order to capture possible rater uncertainty brought on by facial weakness subtlety. The rating scale ranged from 1 to 5 equating to Pathology is: 1) Likely absent, 2) Somewhat likely absent, 3) Indeterminate, 4) Somewhat likely present, and 5) Likely present. If the neurologist rated a video a 4 or 5, then he or she was asked to denote the laterality or side of the facial weakness as either “Left” or “Right.”

After the initial rating of each video by the vascular neurologists, we collapsed the facial weakness ratings down to: 1) Absent, 2) Indeterminate, and 3) Present. This allowed us to establish the “ground truth” that facial weakness is absent, present, or unknown as the mode (most common) of the ratings of the three vascular neurologists. The same manner was used for laterality of facial weakness. This approach is equivalent to majority voting which we believe to be better approach for determine the presence of facial weakness than the traditional approach of using the NIH stroke scale facial palsy item which has a reported interrater reliability kappa as low as 0.25 (5). To note, none of the ground truth ratings of fascial weakness or laterality received a final rating of “unknown"; thus all fascial video had a ground truth rating of absent or present and if present, then laterality was either left or right.

Three blinded paramedics independently classified the videos using the same protocol as the vascular neurologists. The relative experience of the three paramedics included an EMT with 7 years of experience total and 5 years of experience as an advanced life support provider, a nationally registered paramedic with over 10 years of experience, and an entry level EMT with 1 year of experience.

### Computer Vision Algorithm

**Figure 1:**
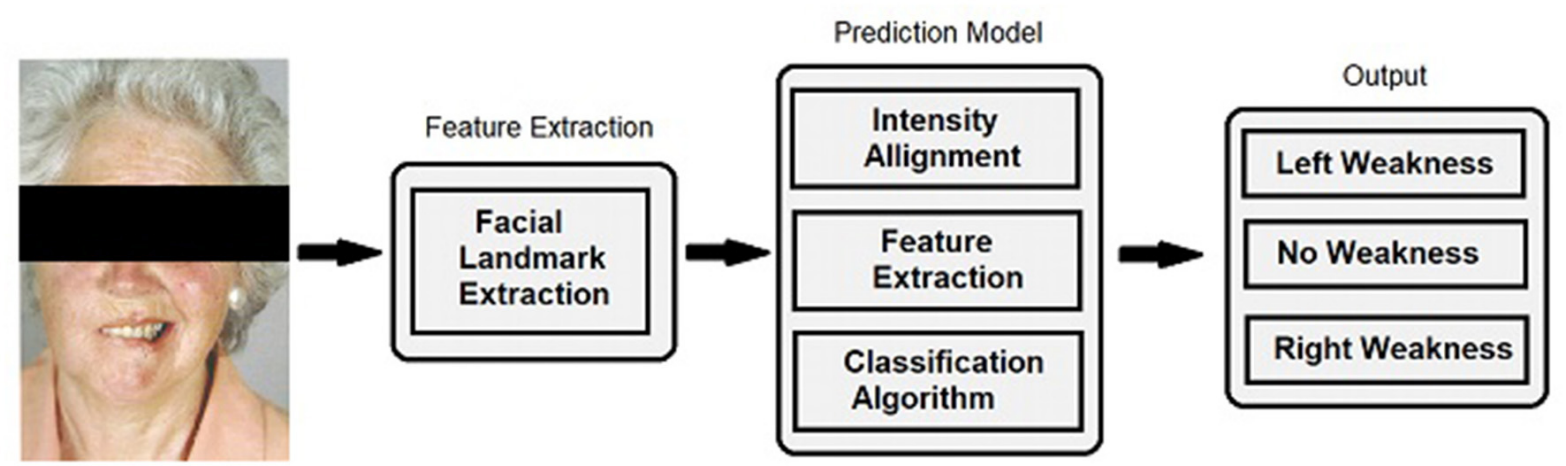
Facial landmark extraction and classification. For algorithm training and performance, videos were decomposed into individual frames and facial landmarks were extracted. Normalization was performed by aligning the extracted landmarks of current input sequences to a template to handle different head scales, location, and orientation. The same transformation was applied to the pixel intensity information to remove these variations. A predictive model computed the classification result for each individual frame and a voting classifier reduced the output into discrete classifications comprised of no weakness, left facial weakness, and right facial weakness categories.

The computer vision algorithm aims to classify the input video as normal, left deficit, and right deficit, by exploiting the Histogram of Oriented Gradients (HoG) (15) feature sets and the penalized Linear Discriminant Analysis (pLDA) technique (16). Detailed description of the algorithm is described in Zhuang et al. (14). To be specific, given a given input video, the framework decomposes the video into a sequence of individual frames, extracts the corresponding facial landmarks, and performs face normalization to remove different translation, scaling, and rotation variations. After preprocessing the video, the HoG features are extracted for each individual frame. We prefer the HoG features over landmarks features, which are commonly done for vision based facial weakness analysis, due to the fact that landmark-based methods can suffer from inaccuracies in face landmarks localization (17, 18), while the HoG features are able to handle local misalignment and capture the detailed gradient features exhibited by facial weakness (15). Since HoG features are high-dimensional, to increase computation efficiency and avoid overfitting, the principal component coefficients are computed from the training dataset to reduce the dimensions of the HoG features to the components that can cover 95% of the variance. Using the principal component coefficient of HoG features as the input, then a supervised pLDA predictive model classifies each individual frame by searching the most discriminant information related to facial weakness. One prominent advantage of the pLDA approach is that it provides visualizable and interpretable results. A detailed formulation and discussion regarding modeling the pathological meaningful texture variations for facial weakness using the pLDA predictive model can be found in Zhuang et al. (17, 19). In the current study, we evaluated the computer vision algorithm developed in Zhuang et al. (14) on a board-certified neurologist verified video dataset used in Zhuang et al. (19), specifically focusing on the comparison between human raters' assessment and the performance of computer vision algorithm from the clinical perspective. Finally, a voting classifier aggregates the individual classification results and outputs discrete classification results: normal, left facial weakness, and right facial weakness (Figure 1). In addition, an ensemble of regression trees based facial landmark extractor (20) is used in our study because of its accurate and robust performance (18). The configurations for HoG features are set as follows: the number of orientation bins in a cell is nine, a cell consists of eight by eight pixels, and each block contains four cells in each block is four. Performance of the algorithm was tested using a 5-fold cross-validation scheme and calculating sensitivity, specificity, and accuracy. The dataset was randomly divided into five groups with balanced samples. Four groups of samples were used for the training process and one group served as the testing dataset. The process was then repeated five times.

### Data Analysis

For the paramedic ratings, individual and the group average sensitivity, specificity, and accuracy were obtained. A false negative was defined as either 1) the failure to identify right facial weakness (3R) when right facial weakness was the ground truth, or 2) the failure to identify left facial weakness (3L) when left facial weakness was the ground truth. A false positive was defined as denoting the presence of facial weakness when normal smile was the ground truth. The algorithm and paramedic groups' sensitivity, specificity, and accuracy were compared and confidence intervals were constructed by 10,000 bootstraps at the patient level and balanced between videos with and without facial weakness, which accounts for repeated measures on patients. This method allows for the estimation of variability for each paramedic as well. To further analyze performance, the identification of unilateral facial weakness was divided into two components: 1) identifying the presence of weakness and 2) identifying the correct laterality of the weakness. Laterality designations (L or R) were initially excluded from video classification. To assess identification of correct laterality, we looked at all reviews where an error was made, and compared whether the rater was human or not with whether or not the error was in laterality using Fisher's exact test with a *p*-value threshold of 0.05 to assign significance.

### Comparison to Prior Work

The present study utilizes the same data set from Zhuang et al. (14), detailed in Section 2.2, and a similar rating schema described in Zhuang et al. (19), in order to compare the current algorithm's performance in detecting facial weakness to trained paramedics. In contrast to these prior studies, the current analysis explores the variability among paramedics and how this variability, as seen in real life practice, compares to the algorithm's performance. We also seek to determine the accuracy of identifying the correct laterality of facial weakness by the algorithm vs. paramedics, which is a key skill in evaluating patients with facial weakness in clinical practice.

## Results

### Demographics

A total of 202 videos were collected: 13 videos were excluded due to failure to detect facial landmarks due to variations in lighting and head position. Of the 189 remaining videos (117 females), 96 demonstrated a normal smile (58% women), and 93 demonstrated unilateral facial weakness (61% women). Of the videos with facial weakness, 50 were with right sided weakness and 43 with left sided weakness. Based on skin-tone, 155 videos had light-skinned individuals and 34 individuals were dark-skinned. All videos were standardized to a rate of 30 frames per second. Overall the videos had a median (range) number of frames of 60 (12 to 260). Videos with normal smiles had a median (range) number of frames 48 (13 to 157), while those with facial weakness had a median of 79 (23 to 260).

Accurate information on age, race, and ethnicity from the videos was not possible to obtain. However, descriptively the videos exhibited a wide range of facial characteristics including glasses, beards, tattoos, and cultural-specific facial painting or adornment. The faces of the individuals ranged from adolescent to elderly in appearance. Solely based on visual inspection to assess ethnicity, the videos encompassed a multi-ethnic sample including people of African, Europe, and South-East Asia ancestry. To provide information about the degree of difficulty of rating the videos for the board-certified vascular neurologists, we calculated the Fleiss Kappa as a measure of agreement (21). The neurologists showed almost perfect agreement (0.8–1.0) for the presence of facial weakness (0.90), for laterality of weakness (0.91), and combined (0.89).

### Algorithm and Paramedic Diagnostic Comparison

As previously reported, the paramedics had a mean accuracy, sensitivity and specificity of 92.6% [95% CI 90.1–94.7%], 87.8% [95% CI 83.9–91.7%] and 99.3% [95% CI 98.2–100%], respectively (19). This study's algorithm had an accuracy, sensitivity and specificity of 88.9% [95% CI 83.5–93%], 90.3% [95% CI 82.4–95.5%] and 87.5 [95% CI 79.2–93.4%]; see Table 1. While there was no difference in accuracy and sensitivity between the paramedics and the algorithm, paramedic assessments were more specific. Overall, the performance of the algorithm to detect the presence of facial weakness was similar to paramedics (*p* = 0.074).

**Table 1:** Performance metrics of the correct identification of facial weakness and its laterality among all raters.

**Facial weakness detection performance**						
	**Paramedics Overall**		**Algorithm**		**ZeroR**	
	**Estimate**	**95% CI**	**Estimate**	**95% CI**	**Estimate**	
Accuracy	92.60%	90.1–94.7%	88.90%	83.5–93%	49%	
Sensitivity	87.80%	83.9–91.7%	90.30%	82.4–95.5%	100%	
Specificity	99.30%	98.2–100%	87.50%	79.2–93.4%	0%	
	**Paramedic 1**		**Paramedic 2**		**Paramedic 3**	
	**Estimate**	**95% CI**	**Estimate**	**95% CI**	**Estimate**	**95% CI**
Accuracy	94.70%	91.5–97.9%	94.20%	90.5–97.4%	88.90%	84.1–93.1%
Sensitivity	95.70%	91–99%	89.20%	82.6–95%	78.50%	69.9–86.5%
Specificity	93.80%	88.5–98%	99.00%	96.6–100%	98.90%	96.6–100%

*95% Confidence intervals are the result of 10,000 boot-straps via the percentile method. ZeroR shows*.

*the expected diagnostic performance based on random selection of the presence of facial weakness*.

**Figure 2:**
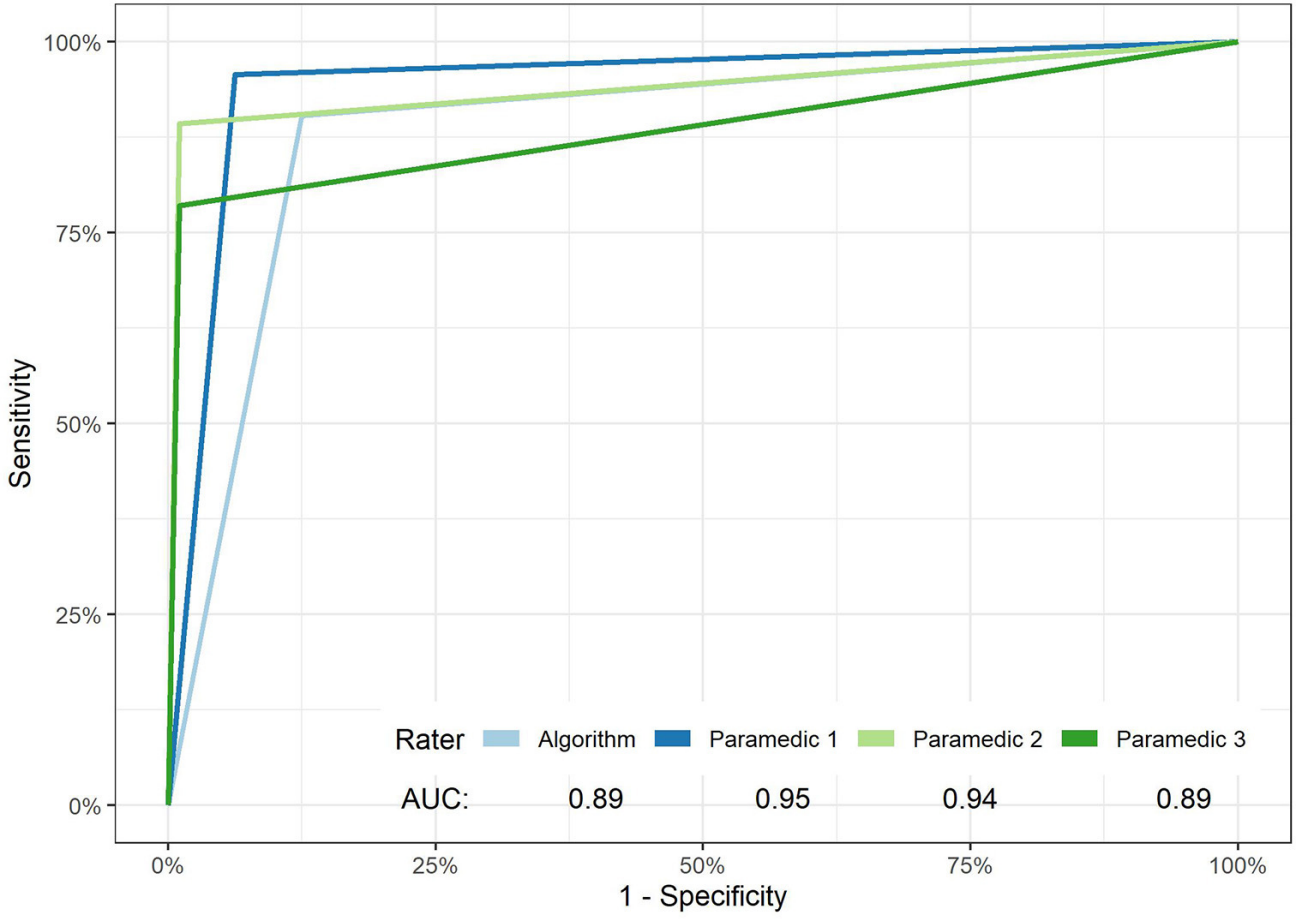
ROC curves of facial weakness and laterality detection of the algorithm and each paramedic. The overall performance of paramedics vs. the algorithm in the detection of unilateral facial weakness failed to reach significance (*p*-value 0.074).

On the other hand, the paramedic group showed notable variability in the sensitivity of facial weakness detection, Table 1. Paramedic sensitivity ranged from 78.5% (69.9–86.5%) to 95.70% (97–99%), a large 17.2% difference. This was not the case for specificity which had a non-significant 6.2% point difference. Figure 2 exhibits the ROC curves for the algorithm and each paramedic.

As a sensitivity analysis, we excluded laterality designations. This did not change the performance metrics of the algorithm. In contrast, the exclusion of laterality improved the average paramedic sensitivity to 92.5 [89.5–95.2%] from 87.8%, but not for specificity. Paramedics made more laterality errors than the algorithm (Table 2) (13 vs. 0, *p*-value = 0.044).

**Table 2:** Cross tabulation ratings of presence and laterality of unilateral facial weakness by paramedics as a group vs. the machine learning algorithm.

	**Presence of unilateral facial weakness**		
	**Present**	**Absent**	**Total (*n* = 189)**
**Paramedics**			
Present	82	1	83
Absent	11	95	106
**Algorithm**			
Present	84	12	96
Absent	9	84	93
	**Side of facial weakness**		
	**Correct laterality**	**Incorrect laterality**	
Paramedics	245	13	
Algorithm	84	0	

*Three independent and blinded board-certified vascular neurologists determined the ground truth labeling for the presence or absence of unilateral facial weakness and the affected side of the face. For the presence of facial weakness, there was no significant differences between Paramedics (as a group) and the algorithm, p-value 0.074. The Paramedics, however, had significantly more laterality errors compared to the algorithm, p-value 0.044. Both comparisons utilized Fisher's exact test*.

## Discussion

In this preliminary study, we demonstrate that a machine learning algorithm using computer vision analysis can detect facial weakness in recorded videos with an accuracy and sensitivity comparable to trained paramedics. However, the average paramedic ratings had better specificity than the algorithm (99 vs. 88%). Just last year, our team has shown that this approach can achieve higher accuracy (94.3%), sensitivity (91.4%), and specificity (95.7%) performance in comparison to paramedics (19), especially when juxtaposing the enhanced algorithm to several state-of-the art methods. Unlike our previous work (19), we show in this analysis the inter-rater variability among paramedics; see Table 1 and Figure 2. The variability in performance among the paramedic was related to differences in sensitivity. This may reflect real-life interactions between paramedics and patients suffering from a stroke in the field. Paramedics have varying years on the job, training programs, and local stroke incidence rates that affect their individual ability to detect stroke. To further this point, Brandler and colleagues observed that ambulance-based paramedics had 83% sensitivity and 66% specificity to detect facial weakness (6). When laterality designations were excluded in our sensitivity analysis, we found that EMS provider ratings had an average sensitivity and specificity of 92.5 and 99%, respectively. The higher specificity as compared to sensitivity may be due, in part, to our pre-specified definitions of false negatives and false positives. Accordingly, laterality errors were designated as false negatives rather than false positives. Given that the EMS providers made several laterality errors, these errors decreased the sensitivity but not the specificity of the paramedic group. However, we did not make this comparison with our updated algorithm (19). It is encouraging that our algorithm's facial detection accuracy (89%) can be improved to 94.5% (19).

An interesting note, the algorithm made no laterality errors compared to paramedics. A possible explanation is that human examiners may make two independent decisions: 1) a more global assessment of facial asymmetry and the presence of pathology followed by 2) closer inspection and selection of the side that appears abnormal. There is no evidence of such dissociation for computer-based detection of unilateral facial weakness. More research is needed to further explore this hypothesis. As a pilot observational study, there are several limitations to consider. As a publically available repository, the dataset was quite heterogeneous with varying video length, quality and formatting. Given the lack of available medical information and dedicated exam maneuvers, particularly to assess the upper facial muscles, we did not seek to classify facial weakness as peripheral vs. central in origin (e.g., Bell's palsy vs. stroke). The inclusion of both younger individuals with peripheral patterns of facial weakness and older individuals with no visible evidence of upper facial weakness suggests that multiple disease processes are represented in the dataset. While patient heterogeneity may broaden the generalizability of the algorithm, further validation in well-curated datasets and live patient encounters is underway.

Another limiting factor of our study is that sensitivity and specificity of the paramedic assessments were higher than expected and much more so than reported by Brandler and colleagues (6). One possible explanation is that our video dataset included an overrepresentation of more severe and obvious cases of facial weakness, as individuals are more likely to upload extreme examples rather than subtle facial asymmetry. In real world practice, many stroke patients will present with more subtle facial weakness, indicated by flattening of the nasolabial fold or slight asymmetry in facial expressions. In these incidences, a computer algorithm applying quantifiable landmark extraction, rather than gross visual inspection, might perform better by comparison.

Although the landmarks extraction approach performed well in terms of localization accuracy and computation efficiency (17), approximately 6% of videos were excluded from analysis due to failure of the algorithm to detect landmarks. The reasons are likely 2-fold. First, we reported a previous study (17) suggesting that the accuracy of the facial landmark extraction approach is insufficient in some cases, where the patients demonstrate severe facial weakness symptoms. This is because modern landmarks detection systems are typically trained and calibrated using normal facial configuration while facial weakness subjects may demonstrate a more pathological configuration. Second, performing facial landmarks detection in an uncontrolled dataset, such as our publically curated videos, is a challenging task (22). Variations arise not only from different denvironmental settings (e.g., indoor vs. outdoor), but also from individual appearance variations (e.g., glasses, mustaches, wrinkles, makeup). This is why we sampled a diverse set of public videos for this study as detailed in the results section. These factors contribute to variability in facial landmarks detection, but they are representative of the same issues one would encounter with real patients in the emergency setting.

Future approaches will seek to first train a dedicated facial landmark extractor for real world patients with facial weakness, and second to explore whether an interactive interface that gives corrective commands to the user can decrease such errors by limiting variability in variables such as head position and lighting.

The ability to detect facial weakness through computer vision analysis is a proof of concept that computer vision methods can be applied to detect other visually observable neurologic signs that could help specify not only stroke, but various stroke subtypes such as large vessel occlusions (e.g., gaze preference, hemiplegia, neglect) or posterior circulation stroke (e.g., nystagmus, dysconjugacy, limb ataxia). Further, we believe machine learning techniques could be applied to non-visual neurologic signs such as aphasia and dysarthria through alternative means such as natural language auditory processing. In a future state, our goal would be to integrate these ML algorithms into an automated version of the NIHSS (i.e., eNIHSS) that could be deployed on mobile devices and expedite the accurate diagnosis and differentiation of stroke for non-neurology providers. The greatest potential for a future at scale implementation of this technology could be the integration into telemedicine or telestroke consults. This possibility is especially poignant due to the rapid advancement and acceptance of telemedicine technology in recent years.

## Summary/Conclusions

This study offers initial proof-of-principle that a computer vision machine learning algorithm can detect lateralized facial weakness with similar accuracy to trained paramedics. We are currently applying similar methods for automated detection of other focal neurological signs common in stroke. External validation is needed in independent datasets and prospective patient encounters.

## Data Availability Statement

The raw data supporting the conclusions of this article will be made available by the authors, without undue reservation.

## Ethics Statement

The studies involving human participants were reviewed and approved by University of Virginia Internal Review Board (IRB). Written informed consent for participation was not required for this study in accordance with the national legislation and the institutional requirements.

## Author Contributions

CA: drafting/revision of the manuscript for content, including medical writing for content, analysis or interpretation of data, additional contributions, manuscript preparation, and formatting for submission. MM: drafting/revision of the manuscript for content, including medical writing for content, major role in the acquisition of data, study concept or design, and analysis or interpretation of data. MW: drafting/revision of the manuscript for content and including medical writing for content and major role in the acquisition of data. YZ: drafting/revision of the manuscript for content, including medical writing for content and analysis or interpretation of the data. OU: drafting/revision of the manuscript for content, including medical writing for content, study concept or design and analysis or interpretation of data. TM: analysis or interpretation of data. IL: major role in the acquisition of data. HP and BS: major role in the acquisition of data. WD, JC, SC, and BW: drafting/revision of the manuscript for content and including medical writing for content. GR: drafting/revision of the manuscript for content, including medical writing for content, study concept or design, analysis or interpretation of data. AS: drafting/revision of the manuscript for content, including medical writing for content, major role in the acquisition of data, study concept or design, and analysis or interpretation of data. All authors contributed to the article and approved the submitted version.

## Funding

This study was supported by the UVA Coulter Translational Research Fund and the UVA Medical Student Summer Research Program.

## Conflict of Interest

MM and OU disclose ownership interest in Neuroview Diagnostics, LLC. Neuroview Diagnostics played no role in study funding, data collection, or data analysis. AS and GR served as unpaid scientific advisors for Neuroview Diagnostics, LLC. AS receives research support for a prehospital stroke trial from Diffusion Pharmaceuticals, Inc. MM, YZ, OU, AS, and GR disclose U.S. Provisional Patent Application No. 62/620,096 and International Patent Application No. PCT/US19/14605. BBW serves as Deputy Editor of the journal, Neurology. AS is past section Editor of the Neurology podcast. The remaining authors declare that the research was conducted in the absence of any commercial or financial relationships that could be construed as a potential conflict of interest.

## Publisher's Note

All claims expressed in this article are solely those of the authors and do not necessarily represent those of their affiliated organizations, or those of the publisher, the editors and the reviewers. Any product that may be evaluated in this article, or claim that may be made by its manufacturer, is not guaranteed or endorsed by the publisher.
